# Correction: Nanochannel conduction in piezoelectric polymeric membrane using swift heavy ions and nanoclay

**DOI:** 10.1039/d2ra90012b

**Published:** 2022-02-25

**Authors:** Karun Kumar Jana, Niraj Kumar Vishwakarma, Biswajit Ray, Saif A. Khan, Devesh K. Avasthi, Manjusri Misra, Pralay Maiti

**Affiliations:** School of Materials Science and Technology, Indian Institute of Technology, Banaras Hindu University Varanasi 221 005 India pmaiti.mst@itbhu.ac.in; Department of Chemistry, Banaras Hindu University Varanasi 221 005 India; Inter University Accelerator Centre Aruna Asaf Ali Marg New Delhi 110 067 India; School of Engineering and the Department of Plant Agriculture, University of Guelph Thornbrough Building Guelph Ontario NIG2W1 Canada

## Abstract

Correction for ‘Nanochannel conduction in piezoelectric polymeric membrane using swift heavy ions and nanoclay’ by Karun Kumar Jana *et al.*, *RSC Adv.*, 2013, **3**, 6147–6159. DOI: 10.1039/C3RA23176C

The authors regret a mistake in Fig. 1a and in the caption of Fig. 1a. Fig. 1a shows SEM images of PVDF and NH surfaces after etching at different fluences. In Fig. 1a of the original manuscript, the panel for PVDF after etching at 1 × 10^6^ fluences incorrectly showed a zoomed in section of the panel of PVDF after etching at 1 × 10^5^ fluences.

**Fig. 1:**
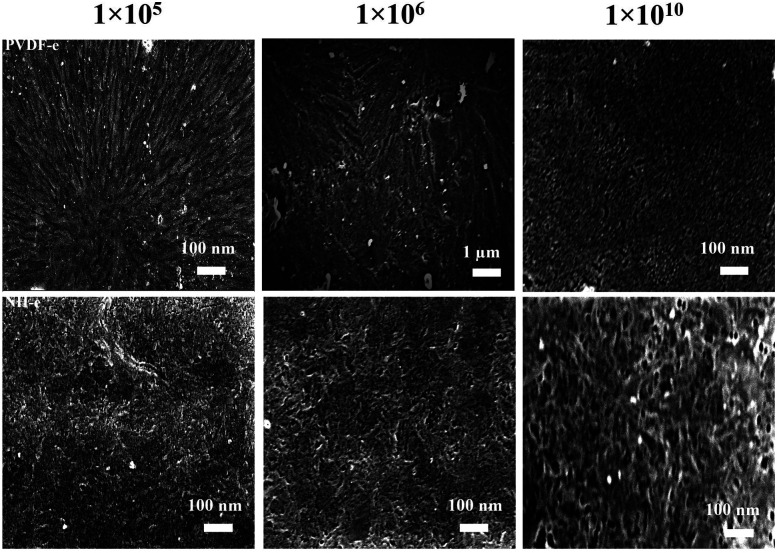
(a) SEM images of the PVDF (upper row) and NH surfaces (lower row) after etching at different fluences.

The caption of Fig. 1a in the original manuscript incorrectly described the SEM images of PVDF to be shown in the upper column and NH surfaces to be the lower column.

The new figure has been reviewed by an expert and is provided here with the corrected caption. This correction does not alter the conclusions presented in this *RSC Advances* paper.

The Royal Society of Chemistry apologises for these errors and any consequent inconvenience to authors and readers.

## Supplementary Material

